# Supplementary material for: Corallococcus soli sp. Nov., a Soil Myxobacterium Isolated from Subtropical Climate, Chalus County, Iran, and Its Potential to Produce Secondary Metabolites
Source: Microorganisms. 2022 Jun 21;10(7):1262. doi: 10.3390/microorganisms10071262 (PMC9323933; doi:10.3390/microorganisms10071262)
Supplement: Supplementary file 1 [file microorganisms-10-01262-s001.zip › microorganisms-1681850-supplementary.pdf]

Supplementary Material (Table)

Corallococcus soli sp. nov., a soil myxobacterium isolated from subtropical climate, Chalus county, Iran and its potential to produce secondary metabolites

Zahra Khosravi Babadi[1,2*]†, Ronald Garcia[3,4]†, Gholam Hossein Ebrahimipour[1], Chandra Risdian[2,5]†, Peter Kämpfer[6], Michael Jarek[7], Rolf Müller[3,4] and Joachim Wink[*2]

1 Department of Microbiology and Microbial Biotechnology, Faculty of Life Sciences and Biotechnology, Shahid Beheshti University GC, Tehran, Iran; Zahrakhosravi1365@yahoo.com; G-ebrahimi@sbu.ac.ir

2 Microbial Strain Collection, Helmholtz Centre for Infection Research (HZI), Inhoffenstraße 7, D-38124 Braunschweig, Germany; Joachim.Wink@helmholtz-hzi.de; Chandra.Risdian@helmholtz-hzi.de

3 Helmholtz Institute for Pharmaceutical Research Saarland (HIPS), Helmholtz Centre for Infection Research (HZI), Saarland University, Campus E8 1, 66123 Saarbrücken, Germany; ronald.garcia@helmholtz-hips.de; rolf.mueller@helmholtz-hips.de

4 German Centre for Infection Research (DZIF), Partner Site Hannover-Braunschweig, Braunschweig, Germany; ronald.garcia@helmholtz-hips.de; rolf.mueller@helmholtz-hips.de

5 Research Unit for Clean Technology, National Research and Innovation Agency (BRIN), Bandung 40135, Indonesia

6 Department of Applied Microbiology, Justus Liebig University Gießen, 35392 Gießen, Germany; peter.kaempfer@umwelt.uni-giessen.de

7 Genome Analytics, Helmholtz Centre for Infection Research (HZI), Inhoffenstraße 7, D-38124 Braunschweig, Germany; michael.jarek@helmholtz-hzi.de

8 Correspondence: Zahra Khosravi Babadi: Zahrakhosravi1365@yahoo.com; Joachim Wink: Joachim.Wink@helmholtz-hzi.de; Tel.: +49 531 61814223; Fax: +49 531 61819499

† Contributed equally

Table S1: Cellular fatty acid profile of strain ZKHCc1 1396^{T} and all type strains of *Corallococcus* species

The strains include: 1) Strain ZKHCc1 1396^{T}, 2) *Corallococcus exercitus* AB043A^{T}, 3) *C. interemptor* AB047A^{T}, 4) *C. aberystwythensis* AB050A^{T}, 5) *C. praedator* CA031B^{T}, 6) *C. sicarius* CA040B^{T}, 7) *C. carmarthensis* CA043D^{T}, 8) *C. llansteffanensis* CA051B^{T}, 9) *C. terminator* CA054A^{T}, 10) *C. coralloides* DSM 2259^{T}, 11) *C. exiguus* DSM 14696^{T}, and 12) *C. silvisoli* c25j21^{T}. The data for numbers 2-9, 10-11 and 12 were taken from previous studies [1, 2, 3], respectively. Trace amount (tr), < 0.10 %; ND, not determined. Major fatty acid was marked in

**Strain:**	**1**	**2**	**3**	**4**	**5**	**6**	**7**	**8**	**9**	**10**	**11**	**12**
**$C_{10:0}$ (decanoic)**	tr	1.02	2.66	2.15	2.72	0.60	6.15	1.56	1.06	ND	ND	ND
**$C_{11:0}$ (undecanoic)**	ND	2.58	6.56	2.57	1.62	0.36	1.77	1.77	0.96	ND	ND	ND
**$C_{15:0}$ (pentadecanoic)**	tr	19.78	11.36	7.60	7.00	5.87	6.47	20.28	13.78	0.10	tr	0.3
***iso*-$C_{15:0}$ (13-methyl-tetradecanoic)**	**15.78**	**45.07**	**37.03**	**46.96**	**58.01**	**67.4**	**41.18**	**43.61**	**53.50**	**33.90**	**36.45**	**51.08**
***iso*-$C_{16:0}$ (14-methyl pentadecanoic)**	5.57	4.15	5.29	ND	2.06	1.60	3.49	1.16	2.36	2.99	1.76	1.1
**$C_{16:1}$ (palmitoleic)**	0.89	6.97	6.65	0.33	5.32	4,95	8.01	7.84	6.77	ND	ND	ND
**$C_{17:0}$ (2-hexyl-cyclopropaneoctanoic)**	ND	ND	6.77	ND	4.79	ND	ND	2.07	ND	ND	ND	ND
***iso*-$C_{17:0}$ (isomargaric)**	9.41	6.20	10.31	7.45	4.29	5.53	8.50	ND	2.26	9.35	10.69	14.4
**$C_{18:1}$ (oleic)**	ND	0.34	ND	0.36	ND	0.18	ND	5.49	0.43	ND	ND	ND

bold.

Table S2: Media and ingredients used in this study.

Media	Ingredients/manufacturer
**Middelbrock Broth medium**	Becton, Dickinson and Company, France
**Müller-Hinton Bouillon** (MHB)	Carl Roth GmbH + Co.KG, Germany
**Myc-medium**	1% phytone peptone
(pH 7.0)	1% glucose
	50 mM HEPES (11.8 g l^{-1})
	Distilled. Water
**Myxovirescin-medium**	1% soy peptone
(pH 7.0)	0.005% $CaCl_2$
	0.025% $MgSO_4$
	1 mg l^{-1} $CoCl_2$
	100 mM HEPES (23.8 g l^{-1})
	Distilled Water

**P-medium**	0,2% peptone (Marcor M)<br>0,8% starch (Cerestar)<br>0,4% Probion<br>0,2% yeast extract (Marcor typ 9000)<br>0,1% $CaCl_2$ x $2H_2O$<br>0,1% $MgSO_4$ x $4H_2O$<br>100 mM HEPES (23,8 g l^{-1})<br>8 mg l^{-1} Fe-EDTA pH 7,5
**S-medium**	0.2% glucose<br>0.1% $MgSO_4$ x 7 H_2O<br>0.4% Soy flour<br>0.2% yeast extract<br>0.1% $CaCl_2.2H_2O$<br>50 mM HEPES (11.9 g l^{-1})<br>Fe-EDTA 8 mg l^{-1}
**POL-medium**<br>(pH 7.2)	0.3% probion<br>0.3% starch<br>50 mM HEPES (11.9 g l^{-1})<br>0.7% $Mgso_4.7H_2O$<br>0.5% $CaCl_2.2H_2O$<br>Distilled Water
**CY-medium**<br>(pH 7.2)	0.3% casiston<br>0.1% yeast extract<br>0.1% $CaCl_2$<br>50 mM HEPES (11.8 g l^{-1})<br>Distilled Water
**CY / H-medium**	50% CY- / 50% H-medium
**E-medium**<br>(pH 7.4)	0.4% skimmed milk<br>0.4% soy flour<br>1.0% starch<br>0.1% $MgSO_4$<br>50 mM HEPES (11.9 g l^{-1})<br>8 mg l^{-1} Fe-EDTA<br>0.5% glycerine<br>Distilled water
**H-medium**<br>(pH 7.4)	0.2% soy flour<br>0.2% glucose<br>0.8% starch<br>0.2% yeast extract<br>0.1% $CaCl_2$<br>0.1% $MgSO_4$<br>50 mM HEPES (11.8 g l^{-1})<br>8 mg l^{-1} Fe-EDTA<br>Distilled water

Supplementary References

1. Livingstone, P.G.; Ingleby, O.; Girdwood, S.; Cookson, A.R.; Morphew, R.M.; Whitworth, D.E. Predatory organisms with untapped biosynthetic potential: descriptions of novel *Corallococcus* species *C. aberystwythensis* sp. nov., *C. carmarthensis* sp. nov., *C. exercitus* sp. nov., *C. interemptor* sp. nov., *C. llansteffanensis* sp. nov., *C. praedator* sp. nov., *C. sicarius* sp. nov., and *C. terminator* sp. nov. *Appl. Environ. Microbiol.* **2020**. 86, 01931–19.
2. Garcia, R.; Pistorius, D.; Stadler, M.; Müller, R. Fatty acid-related phylogeny of myxobacteria as an approach to discover polyunsaturated omega-3/6 fatty acids. *J Bacteriol.* **2011**. 193, 1930–1942.

3. Zhang, X. J.; Feng, G. D.; Liu, Y.; Zhou, Y.; Deng, X.; Yao, Q.; Zhu, H. *Corallococcus silvisoli* sp. nov., a novel myxobacterium isolated from subtropical forest soil. *Arch. Microbiol*. **2022**. 204, 1-7.
